# The Australian evidence-based clinical practice guideline for attention deficit hyperactivity disorder

**DOI:** 10.1177/00048674231166329

**Published:** 2023-05-30

**Authors:** Tamara May, Edwina Birch, Karina Chaves, Noel Cranswick, Evelyn Culnane, Jane Delaney, Maddi Derrick, Valsamma Eapen, Chantele Edlington, Daryl Efron, Tatjana Ewais, Ingrid Garner, Michael Gathercole, Karuppiah Jagadheesan, Laura Jobson, John Kramer, Martha Mack, Marie Misso, Cammi Murrup-Stewart, Evan Savage, Emma Sciberras, Bruce Singh, Renee Testa, Lisa Vale, Alyssa Weirman, Edward Petch, Katrina Williams, Mark Bellgrove

**Affiliations:** 1 Department of Paediatrics, Monash University, Clayton, VIC, Australia; 2 ADHD Foundation, Epping, NSW, Australia; 3 Albury Wodonga Health, Albury, NSW, Australia; 4 Clinical Pharmacology Unit, Department of Medicine and Melbourne Children’s Trials Centre, Royal Children’s Hospital, Parkville, Vic, Australia; 5 Murdoch Children’s Research Institute, Parkville Vic, Australia; 6 University of Melbourne, Parkville, VIC, Australia; 7 The Royal Children’s Hospital Melbourne, Parkville, VIC, Australia; 8 Speech Pathology Australia, Melbourne, VIC, Australia; 9 Hobart ADHD Consultants, Bellerive, TAS, Australia; 10 South Western Sydney Local Health District and Ingham Institute for Applied Medical Research, UNSW Sydney, Liverpool, NSW, Australia; 11 Department of Paediatrics, University of Melbourne, Parkville, VIC, Australia; 12 Faculty of Medicine, University of Queensland, Herston, QLD, Australia; 13 School of Medicine and Dentistry, Griffith University, Southport, QLD, Australia; 14 ADHD Guideline Development Group, Melbourne, VIC, Australia; 15 Youth Justice, Department of Community and Justice, Grafton, NSW, Australia; 16 NWAMHS–North West Area Mental Health Services, Melbourne, VIC, Australia; 17 Bi-National ADHD Network Committee, RANZCP - Royal Australian and New Zealand College of Psychiatrists, Melbourne, VIC, Australia; 18 Swinburne University, Melbourne, VIC, Australia; 19 Turner Institute for Brain and Mental Health and School of Psychological Sciences, Monash University, Clayton, VIC, Australia; 20 ADHD, ASD and Neurodiversity Special Interest Group, Faculty of Special Interests, RACGP; 21 Rural Medical School, UNSW Medicine & Health, Coffs Harbour, NSW, Australia; 22 Applied Neuroscience Society of Australasia (ANSA); 23 The Knowledge Synthesis Lab, Melbourne, VIC, Australia; 24 Department of Education, VIC, Australia; 25 School of Psychology, Deakin University, Burwood, VIC, Australia; 26 Department of Psychiatry, The University of Melbourne, Parkville, VIC, Australia; 27 Department of Mental Health, Royal Children’s Hospital, Melbourne, VIC, Australia; 28 Department of Psychology, Monash University, Melbourne, VIC, Australia; 29 Occupational Therapy Australia, Splash Paediatric Therapy, Melbourne, VIC, Australia; 30 Hakea Prison, Department of Justice, Perth, WA, Australia; 31 University of Western Australia, Perth, WA, Australia; 32 Monash Children’s Hospital, Clayton, VIC, Australia; 33 AADPA Australian ADHD Professionals Association, Melbourne, VIC, Australia

**Keywords:** Attention deficit hyperactivity disorder, guideline, neurodevelopmental disorder

## Abstract

**Objective:**

The objective of this article was to provide an overview of the development and recommendations from the Australian evidence-based clinical practice guideline for attention deficit hyperactivity disorder (ADHD). The guideline aims to promote accurate and timely identification and diagnosis, and optimal and consistent treatment of ADHD.

**Methods:**

Development integrated the best available evidence with multidisciplinary clinical expertise and the preferences of those with lived experience, underpinned by the Grading of Recommendations, Assessment, Development, and Evaluation (GRADE) framework. The 23 guideline development group members included psychiatrists, paediatricians, general practitioners, psychologists, speech pathologists, occupational therapists, educators, Indigenous psychologists, and people with a lived experience; with two independent chairs and a methodologist. Where appropriate, evidence reviews from the National Institute for Health and Care Excellence (NICE) 2018 ‘Attention Deficit Hyperactivity Disorder: Diagnosis and Management’ guideline were updated. Fifty prioritised clinical questions were addressed in 14 systematic reviews (new and updated from NICE 2018) and 28 narrative reviews.

**Results:**

The 113 clinical recommendations apply to young children (5 years and under), children, adolescents and adults. They provide guidance for clinicians on identification, screening, diagnosis, multimodal treatment and support, including pharmacological and non-pharmacological interventions. The guideline and supporting information are available online: https://adhdguideline.aadpa.com.au/

**Conclusions:**

The guideline was approved by the National Health and Medical Research Council (NHMRC) of Australia and relevant medical and allied health professional associations. It is anticipated that successful implementation and uptake of the guideline by organisations, health care providers and other professionals will increase delivery of evidence-based treatment and improve health outcomes for the more than 800,000 Australians with ADHD.

## Introduction

Attention deficit hyperactivity disorder (ADHD) is a neurodevelopmental condition with an onset before 12 years of age (American Psychiatric Association, 2013). It is often a lifelong condition with persistence into adulthood in 60–86% of individuals (Cherkasova et al., 2021). The symptoms cause clinically significant difficulties with attention and/or hyperactivity and impulsivity, which are inconsistent with a person’s chronological or developmental age (American Psychiatric Association, 2013; Cherkasova et al., 2021). To meet diagnostic criteria, the symptoms of ADHD must negatively impact areas of functioning such as academic and occupational functioning, family, social and intimate relationships, psychological functioning including self-view and self-esteem, the ability to complete daily living activities and participation in leisure activities. Moreover, symptom presentation must be pervasive and present in two or more settings (American Psychiatric Association, 2013). ADHD occurs in approximately 6–10% of Australian children and adolescents and 2–6% of adults (Graetz et al., 2001; Sawyer et al., 2018). Given these prevalence figures and the current population, it is estimated there are at least 800,000 Australians living with ADHD. The economic and well-being costs of ADHD in Australia are estimated to be $20 billion annually (Deloitte Access Economics, 2019; Sciberras et al., 2022).

To date, there has not been a cross-discipline, evidence-based Australian ADHD clinical guideline approved by Australia’s peak medical research body, the National Health and Medical Research Council (NHMRC). This has resulted in an absence of clear and consistent guidance for organisations and clinicians in the identification, diagnosis and treatment of ADHD across the lifespan. It has also had the unintended consequence of devaluing the lived experience of those with ADHD.

The recently NHMRC-approved Australian evidence-based clinical practice guideline for ADHD (the Australian ADHD guideline) aims to promote accurate and timely diagnosis, and provide guidance on optimal and consistent assessment and treatment of ADHD across the lifespan (AADPA, 2022). The guideline details best-practice for ADHD diagnosis and treatment and outlines a roadmap for ADHD research and policy. It includes a focus on everyday functioning, participation and quality of life for care based on age, gender, culture, setting and geography of people who are living with ADHD, and those who support them. The Australian ADHD guideline was developed through addressing the priorities of people with a lived experience of ADHD, health professionals, educators, and service providers. It integrates the best available evidence with multidisciplinary clinical expertise and consumer preferences to provide clinicians, educators, consumers and policy makers with guidance through 113 clinical recommendations.

## Method

The Australian ADHD Professionals Association (AADPA) was invited by the Australian Government’s Department of Health (Grant Agreement ID: 4-A168GGT) in 2018 to deliver the Support for People Impacted by ADHD Program. Monies from this grant were used to fund the development of the guideline. No other funding was received or used in the development of the guideline. The guideline development process defined by the NHMRC (2016) was closely followed to ensure transparent development and conflict of interest processes.

The Australian ADHD guideline is based, in part, on the evidenced-based UK National Institute for Health and Care Excellence (NICE) guidance on the diagnosis and management of ADHD (NICE, 2018). It was developed by updating the NICE evidence reviews, conducting new evidence or narrative reviews for questions not addressed by NICE, and adopting or adapting the NICE guidance to the Australian context.

The methods used to develop the Australian ADHD guideline were aligned with international gold standard Appraisal of Guidelines for Research & Evaluation (AGREE II) criteria, ADAPTE II, and Grading of Recommendations, Assessment, Development and Evaluation (GRADE) to meet the comprehensive NHMRC criteria for approval of evidence-based guidelines. All methods, administrative documentation and the guideline, all recommendations and the technical report can be found at https://adhdguideline.aadpa.com.au/. Factsheets and other supporting information can also be found at this website.

### Review of existing guidelines

Searches for existing ADHD evidence-based guidelines were conducted by the project’s methodologist, with 25 guidelines published between 2001 and 2018 identified. Only three completed evidence review searches within the previous 5 years. The most current of those, the UK National Institute for Health and Care Excellence 2018 guideline ‘Attention Deficit Hyperactivity Disorder: Diagnosis and Management’ [NICE guideline NG87] (NICE, 2018), covered similar content to the other two (German Association of the Scientific Medical Societies [AWMF], 2017; Kemper et al., 2018), and was therefore selected for adaptation.

### Development of priority clinical questions

Clinical questions were identified by the Australian ADHD Professionals Association (AADPA) in consultation with stakeholders. The preliminary list of clinical questions was refined through a structured prioritisation process conducted by a multidisciplinary group representing a broad range of perspectives including clinicians, academics and people with lived experience of ADHD, conducted over 2 days of face-to-face workshops. Contributors reached consensus on the resulting 50 clinical questions to be addressed by this guideline and the method for answering each (either a new or updated NICE systematic review or narrative review).

### Guideline development group (GDG) and conflict of interest process

The multidisciplinary GDG was convened by inviting people with experience living with ADHD, people caring for people with ADHD, and academics with experience in ADHD, to participate in the development of the guideline. Disciplines represented included psychology, child and adolescent psychiatry, adult psychiatry, paediatrics, general practice, applied neuroscience, speech pathology, occupational therapy, nursing, education, clinical pharmacology, and government and private health services. There were eight content subgroups led by members of the GDG.

A formal process was followed to identify and manage competing interests among GDG members. A Conflict of Interest (COI) was defined as an interest of a member of the GDG that conflicted with, or could be perceived to conflict with, the duties and responsibilities of membership and the process of guideline development. This included any outside interest which could introduce any bias into the decision making of committee members. Potential members were asked to declare any COIs over the 3 years preceding the formation of the group and any arising during guideline development.

Conflicts or potential conflicts were managed by a COI Management Group, which comprised the two GDG Chairs, and an ethicist (who also acted as the independent observer), who did not otherwise participate in the guideline development process. This group operated within the AADPA policy for the Identification and Management of Potential Conflict of Interests, which was developed to align with standard A6 of the NHMRC ‘Procedures and Requirements for Meeting the 2011 NHMRC Standard for Clinical Practice Guidelines’ (NHMRC, 2011). The interests of the Chairs were scrutinised by the independent ethics expert of the COI Management Group and the President of AADPA.

### Evidence review methodology

#### Update of NICE evidence reviews

Where appropriate, evidence reviews in the NICE guideline were updated, with permission. The selection criteria and search methods used in the NICE guideline (NICE, 2018) (https://www.nice.org.uk/guidance/ng87) were adopted and rerun from the NICE 2018 search date specific for each question. Additional identified evidence was tabulated using the same outcomes as NICE, assessed for certainty and GRADE (The GRADE Working Group, 2009), and integrated with the existing NICE evidence.

#### New evidence reviews

Where no evidence review existed in the NICE guideline to address the clinical questions, the patient/population, intervention, comparison and outcomes (PICO) framework was used to explore the components of each question and finalise the selection criteria. These components were used to design the search strategies and to include and exclude studies in the evidence review screening stage. A broad-ranging systematic search strategy for terms related to ADHD was adopted from the NICE guideline (NICE, 2018) (https://www.nice.org.uk/guidance/ng87). It was combined with specific searches tailored for the clinical question according to the selection criteria and PICO framework. The search terms used to identify studies addressing the population of interest were not limited, so that studies addressing people with ADHD in all cultural, geographical and socio-economic backgrounds and settings would be identified by the search. The search strategy was limited to English language articles, and there were no limits on year of publication. Searched databases included Medline, PsycINFO, EBM Review, and EMBASE.

#### Study selection criteria and methodological quality

Studies were screened by the methodologist based on whether they met the PICO selection criteria established a priori. Full articles were retrieved for further assessment if the information in the citation and abstract suggested the study met the selection criteria. Uncertainty was resolved through discussion among the methodologist and the leads of the eight GDG content subgroups. In addition to articles of primary studies, systematic reviews that met benchmark criteria and selection criteria were used if they reported outcomes and data, additional to the highest quality included evidence. Risk of bias for each trial and GRADE quality for the body of evidenced was assessed using criteria developed a priori according to study design as outlined in GRADE.

#### Data extraction and synthesis

Data were extracted from included studies into ‘Characteristics of included studies’ tables with relevant details (AADPA, 2022). To make a summary statement about the effect of the intervention to inform evidence-based recommendations, data were presented in tables, and where appropriate, using statistical methods such as meta-analyses. When participants, interventions, outcome measures and timing of outcome measurements were considered sufficiently similar, the Review Manager 5.3 software was used for meta-analyses.

#### Certainty of the body of evidence using GRADE evidence profiles

A GRADE evidence profile was prepared for each comparison within each clinical question, listed by outcome. For each prioritised outcome, a certainty rating was determined based on consideration of the number and design of studies addressing the outcome, and on judgements about the risk of bias of the studies and/or synthesised evidence, inconsistency, indirectness, imprecision and any other considerations that may have influenced the quality/certainty of the evidence. This overall quality/certainty of evidence reflected the extent to which the confidence in an estimate of the effect was adequate to support a particular recommendation (The GRADE Working Group, 2009).

All search strategies, PRISMA flow charts, and results for new and updated searches can be found in the guideline Technical Report (https://adhdguideline.aadpa.com.au/about/technical-report/).

### Narrative reviews

Narrative reviews were completed where questions were less well suited to a systematic evidence review format, and where there was insufficient evidence identified for a question where an evidence review was conducted. Narrative reviews were prepared by small groups of GDG members according to their content expertise. Reviews included key information to answer the clinical question and to guide the GDG to draft clinical consensus recommendations (CCR) or clinical practice points (CPP) and were informed by published research and clinical experience.

### Recommendation development

Evidence-based recommendations were underpinned by the GRADE framework. This considers the volume and quality of evidence informing a recommendation, and the feasibility, acceptability, applicability, cost and implementation considerations of the recommendation.

Specific, unambiguous, actionable recommendations were drafted by the GDG taking into consideration evidence certainty, relevance to the Australian population, the balance of benefits and harms, the values and preferences of the community and clinicians, resource implications, and feasibility and fairness, using the GRADE framework. Three types of recommendations were made, as described in Table 1. Recommendation wording reflected the GDG overall interpretation of the evidence and other considerations. The word ‘should’ indicates the GDG’s judgement that the benefits of the recommended action exceed the harms. ‘Could’ indicates that the quality of evidence was limited, the available studies did not clearly demonstrate advantage of one approach over another, or the balance of benefits to harm was unclear. ‘Should not’ indicates either a lack of appropriate evidence, or that the harms were judged to outweigh the benefits.

**Table 1.** Recommendation types.

EBR	Evidence-based recommendation: a structured/systematic evidence review was performed to answer a prioritised question to inform the recommendation.
CCR	Clinical consensus recommendation: recommendation was developed in either of the following ways: • Evidence to answer a prioritised question was sought, but there was insufficient evidence to inform an EBR. Therefore, a narrative review was prepared by an expert subgroup of the guideline development group (GDG) • For questions of lower priority, or where high-quality evidence is known to be limited or non-existent, evidence was not sought and an expert subgroup within the GDG prepared a narrative review.
CPP	Clinical practice point: guidance based on expert opinion and clinical experience, provided on important issues arising from discussion of evidence-based or clinical consensus recommendations, outside the scope of the evidence-finding process.

EBR: evidence-based recommendations; CCR: clinical consensus recommendations; GDG: guideline development group; CPP: clinical practice points.

The 50 prioritised clinical questions were addressed in 14 systematic reviews (new and updated from NICE, 2018) and 28 narrative reviews, generating 113 clinical recommendations and an additional 21 education, service, policy and research recommendations.

### Results

The evidence-based recommendations derived from the evidence reviews can be found in Table 2. The full set of recommendations is available online at: https://adhdguideline.aadpa.com.au

**Table 2.** Australian ADHD guideline evidence-based recommendations.

No	Type	Recommendation	Strength	Certainty
1	Identification			
1.1	High risk groups			
1.1.1	EBR #CCR	Clinicians should be aware that the following groups of children, adolescents, and adults, have an increased prevalence of ADHD, compared with the general population: Children: • In out of home care diagnosed with oppositional defiant disorder or conduct disorder^a^ Children and adolescents: • Diagnosed with anxiety disorders• with epilepsy• with a history of substance abuse^a^ Adults: • With any mental health disorder (including substance use disorders, borderline personality disorder, intermittent explosive disorder, internet addiction, psychotic disorders, binge eating disorder, a gambling disorder^a^)• who experience suicidal behaviour or ideation People of all ages: • With neurodevelopmental disorders including autism spectrum disorder, intellectual disability, tic disorders, language disorders^a^ and specific learning disorders^a^ • Born preterm • With a close family member diagnosed with ADHD^a^ • Born with prenatal exposure to substances including alcohol and other drugs^a^ • With acquired brain injury^a^ • Who are imprisoned^a^ • With low birth weight^a^ • With anxiety, depressive or bipolar and related disorders^a^ • With sleep disorders^a^	****	⊕⊕ⵔⵔ LOW to ⊕⊕⊕⊕ HIGH
4	Non-pharmacological interventions			
4.2	Parent/family training			
	Young children (under 5 years of age)			
4.2.1	EBR	Parent/family training should be offered to parents/families of young children with ADHD.	****	⊕⊕ⵔⵔ LOW to ⊕⊕⊕ⵔ Moderate
	Children and adolescents (aged 5–17 years)			
4.2.2	EBR	Parent/family training should be offered to parents/families of children with ADHD.	***	⊕⊕ⵔⵔ LOW
4.2.3	EBR	More intensive parent/family training programmes should be offered to parents/families of children with ADHD who have co-occurring oppositional defiant disorder or conduct disorder.	****	⊕⊕⊕ⵔ Moderate
	Cognitive-behavioural interventions			
	Children and adolescents aged 5–17 years			
4.2.8	EBR	Cognitive-behavioural interventions could be offered to children with ADHD.	***	⊕⊕ⵔⵔ LOW
4.2.9	EBR	Cognitive-behavioural interventions should be offered to adolescents with ADHD.	***	⊕⊕ⵔⵔ LOW
	Adults (aged 18 years and above)			
4.2.11	EBR	Cognitive-behavioural interventions should be offered to adults with ADHD.	****	⊕⊕ⵔⵔ LOW
5	Pharmacological interventions			
5.3	Medication choice – children and adolescents (aged 5–17 years)			
5.3.1	EBR	Methylphenidate or dexamfetamine or lisdexamfetamine should be offered as the first-line pharmacological treatment for people with ADHD, where ADHD symptoms are causing significant impairment.	****	⊕⊕ⵔⵔ LOW
5.3.4	EBR	Atomoxetine or guanfacine or clonidine should be offered to children and adolescents if any of the following apply: • Stimulants are contraindicated • The person cannot tolerate methylphenidate, dexamfetamine or lisdexamfetamine • Symptoms have not responded to separate trials of dexamfetamine or lisdexamfetamine, and of methylphenidate, at adequate doses • The clinician considers that the medication may be beneficial as an adjunct to the current regimen Due consideration of risks and safety is required, especially if medications are used in combination	****	⊕⊕ⵔⵔ LOW
5.4	Medication choice – adults (aged 18 years and above)			
5.4.1	EBR	Methylphenidate or dexamfetamine or lisdexamfetamine should be offered as the first-line pharmacological treatment for people with ADHD, where ADHD symptoms are causing significant impairment.	****	⊕⊕ⵔⵔ LOW
5.4.4	EBR	Atomoxetine or guanfacine should be offered to adults with ADHD if any of the following apply: • Stimulants are contraindicated. • They cannot tolerate methylphenidate, lisdexamfetamine or dexamfetamine • Their symptoms have not responded to separate trials of dexamfetamine or lisdexamfetamine and of methylphenidate, at adequate doses • The clinician considers that the medications may be beneficial as an adjunct to the current regimen Due consideration of risks and safety is required, especially if medications are used in combination	****	⊕ⵔⵔⵔ VERY LOW
5.5	Further medication choices			
5.5.1	EBR	The following could be offered to adults with ADHD, in no particular order: • Bupropion • Clonidine • Modafinil • Reboxetine • Venlafaxine Careful monitoring of adverse side effects is required.	***	⊕ⵔⵔⵔ VERY LOW

EBR: evidence-based recommendations; CCR: clinical consensus recommendations; ADHD: attention deficit hyperactivity disorder.

a Indicates a clinician consensus recommendation.

### Background

The ADHD guideline includes a brief background section that covers the clinical features, prevalence, aetiology and outcomes associated with ADHD. The background section also covers information about the course of ADHD and changes across the lifespan, as well as information about co-occurring difficulties associated with ADHD.

### Screening and identification

#### High risk groups

Fifteen studies were found to update the NICE 2018 evidence review. In children and adolescents, 12 different high-risk groups were explored, and 8 had significantly higher risk of having ADHD than the control groups (in order of risk):

- People with autism
- Children in out of home care
- People with epilepsy
- People with intellectual disability
- People with oppositional defiant disorders
- People with anxiety disorders
- People with extremely preterm birth
- People with tic disorders

In adults, nine different high-risk groups were identified and seven of the nine had significantly higher risk of ADHD than the control groups (in order of risk):

- People with borderline personality disorder
- People with Internet addiction
- People with psychotic disorders
- People with substance use disorder
- People with intermittent explosive disorder
- People with a family history of ADHD
- People with suicidal ideation/behaviour

A further narrative review identified additional groups, for example, people with foetal alcohol spectrum disorder (Lange et al., 2018), extremely low birth weight (Momany et al., 2018), eating disorders such as binge eating disorder (Wentz et al., 2005; Yates et al., 2009), sleep disorders (Cortese et al., 2009; Sedky et al., 2014), or problem gambling (Dowling et al., 2015). These groups were not identified in the NICE recommendation, nor were those with suicidal ideation/behaviour and Internet/gambling addictions. The narrative review also identified people with ADHD at risk of not being diagnosed, particularly women and girls (Hinshaw et al., 2022; Quinn and Madhoo, 2014).

##### Recommendations summary

Clinicians *should* be aware that some groups of people are more likely to meet criteria for a diagnosis of ADHD, such as people with a family history of ADHD, people with other neurodevelopmental and mental health conditions and people in some settings, such as in out-of-home care. They *should* be aware that ADHD could be under-recognised in girls and women.

#### Screening

A narrative review was conducted given the existence of a recent systematic review with meta-analysis on this topic (Mulraney et al., 2021). Screening rating scales for ADHD include clinician observation, self-report, parent-report, teacher-report or other informant-report. For children and adolescents, screening tools include (but are not limited to) the Vanderbilt ADHD Diagnostic Rating Scale, Conners’ Rating Scales and Strengths and Difficulties Questionnaires, and for adults the Adult ADHD Self-Report Scale (ASRS) and Wender Utah Rating Scale (WURS).

Mulraney et al. (2021) explored the sensitivity and specificity of screening tools for ADHD in children and adolescents. They found none of the screening tools met acceptable levels of sensitivity and specificity (defined as both over 80%). The meta-analysis comparing high-risk with community-based study populations found no significant difference in sensitivity and specificity.

In adults, there have been mixed findings in screening studies. A study of the ASRS found sensitivity and specificity rates below 80% for the general population (Kessler et al., 2005). Another study of individuals with ADHD and randomly selected controls from the population found both sensitivity and specificity levels at 80% and above for both the ASRS and WURS. There was better performance by the longer WURS than the ASRS for specificity at higher sensitivity levels (Brevik et al., 2020). Other studies of the *DSM*-5 version of the ASRS, the ASRS-5, have found both specificity and sensitivity levels above 80% in non-clinical controls (Baggio et al., 2021; Ustun et al., 2017).

In high-risk groups, sensitivity and specificity have varied. In individuals with major depression, the ASRS-v1.1 showed both specificity and sensitivity below 80% (Dunlop et al., 2018). There was acceptable sensitivity but not specificity in studies of substance use disorders (Daigre Blanco et al., 2009; Van de Glind et al., 2013) and incarcerated women (Konstenius et al., 2015). A modified version of the Barkley Adult ADHD Rating Scale (BAARS-IV) showed sensitivity and specificity levels above 80% in adult prison inmates (Young et al., 2016). Studies of the ASRS-5 found acceptable sensitivity but not specificity in individuals with bipolar disorder and/or borderline personality disorder (Baggio et al., 2021) and other clinical groups (Ustun et al., 2017). Thus, screening measures may have difficulties differentiating adult ADHD from other psychiatric conditions that have similar or overlapping symptoms.

The guideline acknowledged there may be underdiagnosis of ADHD in a range of education (primary, secondary or tertiary), health and correctional settings. However, the recommendations were based on the levels of screening test accuracy noted above and costs/benefits.

##### Recommendations summary

Routine screening for ADHD *should not* occur at the population level (for example, in preschools, primary, secondary schools and universities/TAFEs).

Services and clinicians *should* be aware of the sensitivity and specificity of screening tools used. Positive screening *should* be followed by comprehensive clinical assessment for ADHD.

### Diagnosis

Narrative reviews were completed for making an ADHD diagnosis, co-occurring conditions and differential diagnosis.

#### Assessment and diagnosis

A recent review of the quality of five major international diagnostic guidelines (National Institute for Health and Care Excellence guidelines, Scottish Intercollegiate Guidelines Network, Canadian Attention Deficit Hyperactivity Disorder Resource Alliance [CADDRA], British Association of Psychopharmacology and the American Academy of Paediatrics) reported that all guidelines recommended a categorical diagnosis approach based on the *DSM* or ICD classifications (Razzak et al., 2021). All recommended using interview and questionnaires, as well as multiple informants, as key components of the diagnostic process. Also identified was a CAADRA review of systematic reviews and meta-analyses published between 2006 and 2016 on the diagnosis of ADHD. It found no strategies that achieved additional benefit beyond that of clinician interview in combination with rating scales. Direct observations such as observing children in their educational setting, neuropsychological and psychoeducational assessments, computerised cognitive assessments, neuroimaging and electroencephalography (EEG) did not increase the accuracy of diagnosis. The recommendations for diagnosis were consistent with NICE and CAADRA recommendations.

#### Co-occurring conditions

A high proportion of people with ADHD have co-occurring neurodevelopmental, mental health and medical conditions. In children, the most common co-occurring disorders are oppositional defiant disorder, language disorders, autism spectrum disorders and anxiety disorders, with depressive disorders and substance use disorders emerging in adolescence. Specific learning disorders also commonly occur in people with ADHD and involve difficulties in reading, written expression or mathematics (DuPaul et al., 2013). Among adults with ADHD, the most common co-occurring mental health disorders are depressive disorders, bipolar disorder, anxiety disorders, personality disorders and substance use disorders (Kessler et al., 2006). Medical conditions, such as epilepsy, can co-occur with ADHD (Ilie et al., 2015; Lange et al., 2018). For people with ADHD and a co-occurring condition, the onset, duration and pattern of functional impact may help differentiate the effects of ADHD from those of the other condition, to help guide the treatment plan.

#### Differential diagnosis

Some medical disorders can be present and have symptoms and signs similar to those of ADHD, such as obstructive sleep apnoea/sleep deprivation. Several medications can also produce symptoms similar to those of ADHD, for example, anti-epileptics such as Keppra (American Psychiatric Association, 2013). Clinicians *should* conduct a comprehensive assessment (including history and examination) to identify any possible differential medical causes for ADHD.

In addition to medical conditions, neurodevelopmental and mental health conditions should be considered during differential diagnosis. These disorders may be potentially misdiagnosed as ADHD due to overlapping symptoms and consequences (American Psychiatric Association, 2013). Careful consideration of the onset and course of symptoms is required to make decisions about differential diagnosis. For example, difficulties with concentration and focusing attention that are associated with a major depressive episode are usually limited in duration, whereas attention problems due to ADHD are often ongoing. There are no specific conditions that must be excluded for a diagnosis of ADHD. *DSM*-5 provides further specific advice on differential and co-occurring diagnoses (American Psychiatric Association, 2013).

##### Recommendations summary

A thorough assessment by an appropriately trained clinician is needed to make a diagnosis of ADHD. A person with ADHD may have one or more other neurodevelopmental, mental health or medical conditions that make diagnosis and treatment more complex. Careful assessment of possible co-occurring or alternative conditions is required.

### Information needs after diagnosis

There is no robust research evidence on what information and support should be routinely provided at diagnosis to people with ADHD. Parents of children with ADHD have expressed the need for concise, tailored and reliable information (Ahmed et al., 2014). This includes information on the causes, mechanisms and potential impacts of having ADHD (Ahmed et al., 2014). There is a need to provide information to the person with ADHD, parents, families, education institutions and workplaces about the symptoms and functional impact of ADHD, treatment and support required, and to dispel myths. Given a lack of research in this area, the NICE guideline recommendations were adapted to suit the Australian context.

#### Recommendations summary

After diagnostic assessment, clinicians *should* provide the person with ADHD and their carers with education and information on the causes and potential consequences of ADHD and evidence-based treatments, in a way that instils hope and motivation and focuses on strengths. Information *should* be provided in a format that best suits the unique needs of the person with ADHD. People with ADHD and their families should be provided with information about support and advocacy groups, and financial support such as government disability support and benefits.

### Multimodal treatment

An updated evidence review was conducted and no new evidence was found, as such the recommendations from NICE were accepted and adapted for the Australian context.

#### Recommendations summary

**Figure 1.**
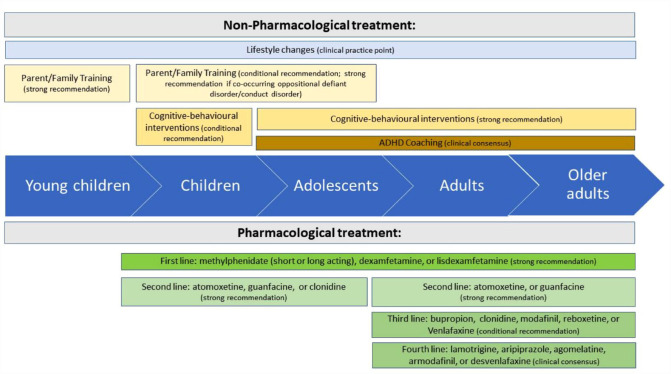
Non-pharmacological and pharmacological treatments.

Clinicians *should* offer multimodal treatment and support. Figure 1 summarises the treatments recommended by the guideline. Clinicians should explain that pharmacological treatment is most effective in reducing core ADHD symptoms and that non-pharmacological treatments provide additional support to minimise the daily impact of ADHD symptoms and associated difficulties (see Table 3). Clinicians *should* describe the typical benefits, adverse effects, efficacy, treatment length, and time taken before symptom or functional improvements occur for each mode of treatment.

**Table 3.** Main targets for pharmacological and non-pharmacological treatment.

	Pharmacological treatment	Non-pharmacological treatment
Primary outcome	Symptom reduction	Improved functioning and wellbeing
Secondary outcomes	Improved functioning and wellbeing	Symptom reduction

The treatment plan and sequence of treatments *should* accommodate the person’s preferences, unique needs and individual goals, and take into consideration their personal strengths and the impact of any co-occurring conditions.

As a child with ADHD grows up, their clinicians *should* plan for a smooth move from health services for children/adolescents to adult health services.

### Non-pharmacological interventions

An updated evidence review was conducted, and an additional 28 randomised controlled trial were identified and included in new GRADE evidence tables (AADPA, 2022). NICE outcomes adopted for the guideline included the primary outcome of ADHD symptoms and secondary outcomes including quality of life, other symptoms (e.g. executive functioning or symptoms of other conditions), functional outcomes, clinical global impression, academic performance, emotion dysregulation and self-harm, as well as adverse events. Additional important secondary outcomes for parent/family training such as parent self-efficacy and family functioning were explored narratively. Non-pharmacological interventions were categorised by type of intervention and included cognitive behavioural interventions delivered via parent/family training or directly to individuals with ADHD; cognitive training; neurofeedback; and organisational/school-based interventions. Narrative reviews were conducted to explore the role of ADHD coaching and peer support workers.

Table 4 and Figure 1 summarise the non-pharmacological interventions.

**Table 4.** Summary of non-pharmacological interventions.

Intervention	Description	Usual delivery	Age group	Outcomes summary	Recommendation	NICE Recommendations	Knowledge gaps
Lifestyle changes	Involves modifying aspects of daily life to improve health and well-being, including diet, exercise or activity levels, and sleep patterns	Person with ADHD Parents/carers Individual format	All	Lifestyle changes have the potential to improve day-to-day functioning for people with ADHD.	Offer guidance on sleep, diet and physical activity levels, including offering strategies and/or referral if needed	As per Australian but no recommendation regarding sleep	Limited research overall. No studies meeting the guideline criteria identified for adults and children under 5 years
Parent / family training	Help parents to optimise parenting skills to meet the additional parenting needs of children and adolescents with ADHD. Includes ADHD-specific and general parenting guidance	Direct to parents/carers; children sometimes involved. Group or individual format	Children under 5 Children Adolescents	Effectiveness of parent/family training varied according to raters (parents, clinicians or teachers), with more benefits evident by parent report. Improvements found in ADHD symptoms and parent/family functioning based on parent-report.	Offer an ADHD-focused group parent-training programme to parents or carers More intensive parent/family support for children with ADHD and co-occurring oppositional defiant disorder or conduct disorder	As per Australian	Limited available research in under-5s on which subgroups of children with ADHD may benefit more or less
Cognitive behaviour therapy	Cognitive and/or behavioural interventions to minimise the day-to-day impacts of ADHD symptoms. Includes psychoeducation, environmental modifications, behaviour modifications and psychological adjustment and cognitive restructuring	Direct to person with ADHD Group or individual format	Children Adolescents Adults	Evidence supports improvement in parent-reported ADHD symptoms in adolescents; improvement for children unclear. Improvement found in self-reported or investigator rated ADHD symptoms in adults.	Cognitive-behavioural interventions should be offered to adults and adolescents with ADHD and could be offered to children	As per Australian	Few studies examined directly delivered cognitive-behavioural interventions for children
Cognitive training	Use of computerised training programmes to improve aspects of cognitive processes such as attention and working memory or cognitive control rather than ADHD symptoms	Person with ADHD Individual format	Children Adolescents Adults	No robust evidence in children & adolescents for improvements in parent-reported overall ADHD symptom severity, or broader functioning or teacher rated ADHD symptoms. Some improvement in parent-reported inattention and hyperactivity symptoms, but from studies of very low and low certainty. Evidence for adults suggested no clear benefit of cognitive training.	None	As per Australian	Only two adult studies met inclusion criteria, both with very low to low certainty. Evidence remains inconclusive.
Neurofeedback	Also known as EEG (electroencephalography) and biofeedback, applies principles of operant conditioning to teach self-modification of cortical electrical activity.	Person with ADHD Individual format	Children Adolescents Adults	Evidence of benefits of neurofeedback over waitlist/usual care for parent- or teacher-reported ADHD was inconsistent in children and adolescents. There were benefits for ADHD inattention symptoms based on parent-report but not teacher or clinician report; and no benefits for parent or teacher-reported ADHD hyperactivity-impulsivity symptoms. In adults, the evidence was inconclusive	None	As per Australian	Evidence remains inconclusive
Organisation and school-based interventions	Usually involve programmes run at school or before/after school care programmes. Studies included components of teaching organisational skills.	Person with ADHD Group format	Children Adolescents	Some evidence of improved parent-reported inattention symptoms.	None	As per Australian	More research required. Organisational skills were not specifically measured in the studies and should be explored in future studies.
ADHD coaching	Shares common elements with cognitive behavioural interventions, including environmental modification and behavioural modification	Person with ADHD Individual format	Adolescents Adults		ADHD coaching could be recommended as part of a treatment plan	None	Varied approaches to coaching are evident in practice, most building on an in-depth or lived experience of ADHD. More robust research needed
Peer support workers	A person who draws on personal and shared experience of ADHD to support others with similar challenges	Person with ADHD Individual format	Adolescents Adults	Insufficient research	None	As per Australian	More research needed

NICE: National Institute for Health and Care Excellence; ADHD: attention deficit hyperactivity disorder.

Regarding parent/family training, the effectiveness varied according to raters (parents, clinicians or teachers), with more benefits evident by parent report. There is limited evidence to suggest improvements in child ADHD symptoms and/or functioning by teacher report, which is not surprising given the focus of parent/family training is on the home context. Parents are typically unblinded when rating outcomes, whereas clinician and teacher ratings can be blinded, which could introduce bias in parent ratings. As such, the quality of the evidence for parent/family training was low to moderate for children under 5 and low for children and adolescents. It is also noted the narrative review which explored additional outcomes of parent family functioning suggested benefits of parent/family training in one or more of these domains, but again through parent ratings.

#### Recommendations summary

Non-pharmacological interventions can improve broader aspects of functioning for individuals with ADHD and/or their families. Clinicians should offer guidance on lifestyle changes, such as promoting a healthy and active lifestyle, including considering sleep patterns, as these have the potential to improve day-to-day functioning. Parent/family training should be offered to parents/carers of children and adolescents with ADHD to support the functioning of the family and child with ADHD. Cognitive-behavioural interventions should be offered to adolescents and adults with ADHD. Making changes in a person’s school, university or workplace can help the person with ADHD succeed. This can include physical changes or educating other people on how to most helpfully interact with the person with ADHD.

### Pharmacological interventions

An updated evidence review was conducted regarding starting, adjusting, and discontinuing pharmacological treatment; however, new studies were not integrated into the NICE findings as their qualitative assessment had reached saturation (i.e. no further themes identified). A narrative review was also conducted.

#### Starting, adjusting and discontinuing treatment recommendations summary

Evidence indicated that before prescribing medication to treat ADHD symptoms, clinicians should carefully assess the person’s general health, including the person’s physical health such as medical history, current medications, height and weight, and conduct a cardiovascular assessment. Clinicians should explain all medication options including potential benefits and side effects. Clinicians and people with ADHD (and/or their parents/carers) should make treatment decisions together. Choice and dosage of medication must be optimised for each person. Clinicians should provide adequate information about the benefits and side effects of medication treatment and address any concerns around long-term effects. The treating clinician should review progress regularly during the dose-titration period. The dose should be titrated against symptoms, level of functioning and adverse effects until the optimal dose has been identified (i.e. the dose at which symptoms are reduced and functional outcomes are improved, with minimal adverse effects).

An updated evidence review of the efficacy of pharmacological treatments found 16 new studies. The same outcomes for non-pharmacological treatments were used with primary outcomes being ADHD symptom reduction and secondary outcomes including improved functioning and quality of life. Evidence was combined for studies of the same design, age range and medication types. There was a paucity of evidence for the effectiveness of medications in children under 5 years of age. As such, no evidence-based recommendation about medication use in this age range was made.

#### Medication choice recommendations summary

An expert in child development and treating ADHD in young children *should* be involved in assessment and treatment decisions. For children, adolescents and adults, evidence showed monotherapy with methylphenidate, lisdexamfetamine or dexamfetamine was associated with a clinically important benefit, compared with placebo or other agents. It was recommended that methylphenidate or dexamfetamine/ lisdexamfetamine *should* be the first-line treatment for children aged 5 years and over, adolescents and adults, given the minimal difference in efficacy and tolerability in these agents. If one medication type or duration of action of stimulant medication is not effective or poorly tolerated, then other stimulant types or duration of action should be trialled before trialling other medications such as non-stimulants. Practice points regarding starting either short- or long-acting medications were made that differed from NICE recommendations, using an informed and shared decision-making approach rather than specifying first-, second- and third-line stimulants.

No new evidence was identified for sequence of pharmacological/non-pharmacological treatment to be offered when the initial treatment is ineffective, inadequate or treatment is not tolerated. If stimulants are not tolerated or are ineffective, atomoxetine, or guanfacine, and additionally clonidine in children and adolescents, *should* be offered as a second-line treatment. Atomoxetine and guanfacine were the non-stimulant drugs with the most convincing evidence. For adults, third-line treatments with very low certainty of evidence based on the evidence review, that *could* be offered included bupropion, clonidine, modafinil, reboxetine and venlafaxine. A practice point based on GDG clinical expertise was made regarding fourth-line treatments for adults that *could* be offered and included lamotrigine, aripiprazole, agomelatine, armodafinil, desvenlafaxine. The third- and fourth-line medications included for adults in the Australian ADHD guideline were not included in the NICE guidance.

There was very little evidence on medication choice for people with ADHD and most co-occurring conditions. However, the available evidence did not suggest a different approach was warranted for a different choice of ADHD medication for people with ADHD and coexisting conditions, but there *should* be careful baseline assessments and consideration of drug interactions, slower titration and more careful monitoring of adverse effects, and regular contact.

No new evidence was found for an updated evidence review exploring whether planned breaks from stimulant medication should be taken. An updated evidence review was conducted for medication monitoring and discontinuation and one new study was identified. Evidence identified concerned inadequate follow‑up and medication review. Limited evidence showed possible worsening of ADHD symptoms on stopping medication but supported a reduction in adverse effects after withdrawal. The importance of assessing the overall benefits and harms of medication should be examined as part of the annual review.

#### Monitoring treatment and discontinuation of treatment recommendations summary

Evidence showed the clinically important differences in sleep disturbance, decreased appetite and weight changes in people with ADHD taking medication and that ongoing monitoring for these unwanted effects should be undertaken. People taking medication for ADHD should be encouraged to monitor and record their adverse effects. Standard symptom and adverse effect rating scales should be used for clinical assessment and throughout the course of treatment. A yearly review with an ADHD specialist is recommended including a comprehensive assessment that revisits the areas discussed when starting treatment and evaluates the effect of current treatment. This helps ensure that decisions around continuing or stopping treatment are fully informed.

Regarding adherence to pharmacological and non-pharmacological treatment, an updated evidence review was conducted with four new studies identified. The evidence highlighted time management and forgetfulness as common barriers to adherence.

#### Medication adherence recommendations summary

Clinicians *should* be aware that the symptoms of ADHD can reduce adherence, for example, forgetting to collect medication and/or organise review appointments to ensure uninterrupted supply of prescriptions.

### Subgroups

Narrative reviews were conducted for three important subgroups identified in the guideline.

#### People in the correctional system

ADHD prevalence is higher in custodial settings than in the general population, estimated to be 5 times higher among youth prisoners and 10 times higher among adult prisoners (Konstenius et al., 2015; Moore et al., 2016; Westmoreland et al., 2010; Young et al., 2015; Young and Thome, 2011). Unidentified and untreated ADHD increases the likelihood of offending, being arrested and incarcerated, being involved with prison incidents and recidivism. Many prison health systems are overstretched and focus their resources on the acutely unwell. There are challenges in identification, assessment and treatment including screening, psychological services, and medication administration, particularly stimulants. If these challenges can be overcome, there are many likely benefits for prisoners, their families, the prison and its staff, the criminal justice system and the community. Recommendations therefore include the provision of screening and treatment opportunities, including coordination and integration of care with community services.

#### Aboriginal and Torres Strait Islander peoples

Currently, there is a lack of research on understanding, identifying, assessing and treating ADHD in Aboriginal and Torres Strait Islander peoples (Loh et al., 2016). This lack of knowledge may result in either over- or under-diagnosis and cause harm to Aboriginal and Torres Strait Islander peoples through stigma or a lack of treatment. For example, there could be misidentification of symptoms that could be otherwise considered as culturally appropriate behaviours. There is a need to provide culturally appropriate and competent care to all. Recommendations include the need to conduct culturally appropriate screening, assessment and treatment of ADHD in Aboriginal and Torres Strait Islander peoples. A strengths-based focus should be employed wherever possible. Clinicians should be aware that ADHD symptom questionnaires and other tools used for screening and assessing ADHD may not be valid in Aboriginal and Torres Strait Islander peoples and should be used with caution. Clinicians should seek the assistance of a cultural interpreter or Aboriginal and Torres Strait Islander health worker.

#### ADHD in people with co-occurring substance use disorders

ADHD is a risk factor for the development of substance use disorders, and people presenting with substance use disorders are at increased risk of ADHD (Groenman et al., 2013; van Emmerik-van Oortmerssen et al., 2012; see also Faraone et al., 2021; Ozgen et al., 2020). Recommendations include the need for those working in mental health settings, and addiction settings to be aware of the high co-occurrence of substance use disorders and ADHD. Clinicians treating people with ADHD in these settings should routinely screen for problematic substance use or substance use disorders, and clinicians treating substance use disorder should routinely screen for ADHD, using best-practice screening questionnaires. Formal diagnosis of substance use disorders in an individual with ADHD, and ADHD in individuals with substance use disorder should follow recommended guidelines for both. Treatment recommendations are also made.

## Conclusions

This is the first multidisciplinary, evidence-based clinical practice guideline for ADHD in Australia to be approved by the NHMRC. It has attempted to balance traditional medical, biopsychosocial and social disability models, to ensure a considered approach to the identification, diagnosis and support of people with ADHD. Through adoption of these recommendations, the guideline aims to improve the experience and health outcomes for the estimated more than 800,000 Australians with ADHD. It is hoped that the guideline will make clinical practice more consistent across Australia by providing clear advice about evidence-based ADHD identification, diagnosis and treatment.

In addition to the clinical recommendations presented here, the guideline makes recommendations for research, service development, professional development and education. This includes recommending that primary care and public mental health services should make diagnosis and treatment available to people of all ages with ADHD. This is underpinned by a need for ADHD training to be added to the curriculum of relevant clinical programmes and be provided to clinicians working in organisations that provide services to people with ADHD, including all public health services (child, adolescent, adult).

It is noteworthy that of the 113 recommendations made in this guideline, a relatively small number (i.e. 12) were evidence-based recommendations. This highlights limitations in the extant research literature of ADHD, wherein there is a lack of appropriately designed, controlled and powered studies that can be drawn upon to make recommendations for best care in response to key questions raised by the ADHD community. It is also noteworthy that the certainty of the evidence underpinning the evidence-based recommendations was more often rated as either low or moderate rather than high. We therefore call upon the ADHD research community to work with clinicians and individuals who have lived experience of ADHD to prioritise research questions and increase and focus their efforts to design high-quality studies. This can advance the scientific evidence-based of ADHD with a view to enhancing clinical practice and improving health outcomes for individuals with ADHD.

In summary, this guideline provides a roadmap not only for ADHD clinical practice, but for research and policy, and highlights opportunities for further improvements in health care, research and policy to come.
